# What can ATP content tell us about Barth syndrome muscle phenotypes?

**DOI:** 10.20517/jtgg.2024.83

**Published:** 2025-01-14

**Authors:** Jeffrey J. Brault, Simon J. Conway

**Affiliations:** 1 Department of Anatomy, Cell Biology & Physiology, Indiana University School of Medicine, Indianapolis, IN 46202, USA.; 2 Herman B. Wells Center for Pediatric Research, Indiana University School of Medicine, Indianapolis, IN 46202, USA.

**Keywords:** Barth syndrome, TAFAZZIN, cardiolipin, striated muscle, mitochondria, adenosine triphosphate, energetics

## Abstract

Adenosine triphosphate (ATP) is the energy currency within all living cells and is involved in many vital biochemical reactions, including cell viability, metabolic status, cell death, intracellular signaling, DNA and RNA synthesis, purinergic signaling, synaptic signaling, active transport, and muscle contraction. Consequently, altered ATP production is frequently viewed as a contributor to both disease pathogenesis and subsequent progression of organ failure. Barth syndrome (BTHS) is an X-linked mitochondrial disease characterized by fatigue, skeletal muscle weakness, cardiomyopathy, neutropenia, and growth delay due to inherited *TAFAZZIN* enzyme mutations. BTHS is widely hypothesized in the literature to be a model of defective mitochondrial ATP production leading to energy deficits. Prior patient data have linked both impaired ATP production and reduced phosphocreatine to ATP ratios (PCr/ATP) in BTHS children and adult hearts and muscles, suggesting a primary role for perturbed energetics. Moreover, although only limited direct measurements of ATP content and ADP/ATP ratio (an indicator of the energy available from ATP hydrolysis) have so far been carried out, analysis of divergent BTHS animal models, cultured cell types, and diverse organs has failed to uncover a unifying understanding of the molecular mechanisms linking TAFAZZIN deficiency to perturbed muscle energetics. This review mainly focuses on the energetics of striated muscle in BTHS mitochondriopathy.

## INTRODUCTION

### Barth syndrome

Barth syndrome (BTHS; OMIM #302060) is a rare X-linked disorder affecting mainly males and is caused by mutations in the phospholipid-lysophospholipid TAFAZZIN transacylase (HGNC:11577) gene^[1]^. The Tafazzin protein is mitochondrially located and plays an important role in both mitochondrial formation and function^[2]^. BTHS is characterized by dilated cardiomyopathy, neutropenia, growth restriction, growth delay, and skeletal myopathy^[3–7]^. As with most mitochondriopathies, there is no cure for BTHS, and patients often succumb to premature death. BTHS patient mortality is thought to be primarily due to cardiomyopathy, which can progress to heart failure and arrythmias^[3,7]^. Additionally, BTHS skeletal myopathy is detectable from birth and causes low muscle tone (hypotonia), as well as muscle weakness leading to motor skill delay (crawling, walking)^[8]^. BTHS boys and men exhibit muscle weakness, extreme fatigue during strenuous physical activity, and eating difficulties^[8–11]^. Despite myopathy being a cardinal feature of BTHS^[12]^ and mitochondrial dysfunction being well described in BTHS, very little is known about the bioenergetic state of muscle in BTHS. Relevant to this literature review, BTHS animal models are considered models for defective mitochondrial ATP production and, thus, for understanding energy deficits.

### Bioenergetics of ATP

The transfer of energy is central to cell survival. Arguably, the most important intracellular energetic intermediate is ATP^[13]^. This is due, at least in part, to the direct transfer of energy from ATP hydrolysis to drive essential cellular functions such as protein synthesis and degradation, active ion transport, and muscle contractions. The amount of available energy from ATP hydrolysis (${\text{DG}}_{\text{ATP}}$) is defined by the Gibb’s free energy equation:

$$\Delta G_{ATP}=DG_{ATP}^{\circ }+RTln\left(\frac{\left[ADP][Pi\right]}{\left[ATP\right]}\right)$$

where $D{G^{\circ }}_{ATP}$ is the free energy of ATP hydrolysis under standard conditions of temperature, pressure, and substrate/product concentrations in solution, R is the gas constant, and T is the temperature in °K^[14]^. An important aspect of this is that the amount of available energy does not depend solely on the concentration of ATP but, instead, is dependent on the ratio of the ATP to ADP and inorganic phosphate (Pi). Said another way, ATP alone is not a sensitive measure of energetic state nor of mitochondrial function^[15]^.

In extracts from non-contracting skeletal muscle, consensus levels for total ATP are ~5–6 mmol/g, for ADP ~0.5 mmol/g, and for AMP ~0.1 mmol/g, although values differ between muscles with different fiber types^[16,17]^. During periods of substantial energy supply/demand mismatch, such as initial stages of intense contractions or hypoxia, ATP changes little while ADP and especially AMP increase substantially in part because of buffering by the near-equilibrium creatine kinase (PCr + ADP ↔ Cr + ATP) and adenylate kinase (ADP + ADP ↔ ATP + AMP) reactions. Prolonged mismatch between energy supply and demand would lead to continuous ATP decline and cell death.

When steady-state changes in ATP are detected, results could be interpreted in two ways. First, a reduction in ATP with an increase in the degradation products ADP, AMP and/or IMP is an indication of severe and/or prolonged mismatch in ATP supply and demand^[18]^. This can occur with intense muscle contractions^[17,19]^ or hypoxia^[20]^. Second, decreases in ATP with a concomitant decrease in ADP and AMP, i.e., a decrease in the total pool of adenine nucleotides (ATP + ADP + AMP), is an indication of a cellular or phenotypic change without a mismatch in energy supply and demand. As examples of ATP differences without mismatch between energy supply and demand, the total pool of adenine nucleotides differs between different cell types^[21]^ and between muscle fiber types^[16,17]^. Further, acceleration of adenine nucleotide degradation by increased AMP deamination in skeletal muscle is sufficient to decrease ATP without changing the ATP/ADP ratio^[22,23]^. Indeed, it is clear that these two measures, the ATP content and ATP/ADP ratio, are regulated independently^[24]^.

### Measuring adenine nucleotides

Quantifying the energetic state in tissues or cells presents several challenges. First, ATP is rather labile and can be quickly degraded during collection or processing^[25–27]^. Second, a single assay (e.g., simply measuring ATP) is not sufficient, given that both ATP and ADP define the energetic state. Third, the measures of ATP and ADP must be quantified in an absolute term (e.g., mmol/g or mM) and cannot be simply normalized to arbitrary values, because arbitrary units would make the calculation of ATP/ADP meaningless.

ATP in muscle can be measured using a variety of assays, including magnetic resonance spectroscopy (MRS)^[28]^, fluorescent reporters^[29]^, luciferase assays^[30,31]^, and high or ultra performance liquid chromatography (HPLC or UPLC)^[32,33]^. These methods have been used for decades, but each has limitations. MRS and fluorescent reporters are generally used *in vivo*, which avoids tissue collection artifacts. However, they are not amenable to absolute quantification measures (only relative) and generally cannot measure ADP directly in tissue^[28]^, which makes validation across studies impossible and may even be misleading. Luciferase assays can be used to quantify ATP directly and are generally performed on extracts. However, ADP cannot be measured directly. UPLC assays are performed on tissue extracts, can measure ATP and ADP (as well as NAD+, NADH, and adenine nucleotide degradation products such as AMP and IMP) simultaneously, and are highly quantitative. Both luciferase and UPLC assays can only be performed on tissue/cell extracts.

### Tafazzin protein and interactions

TAFAZZIN protein contains mitochondrial localization and membrane anchoring domains, as well as a unique hydrophilic domain that may serve as an exposed loop interacting with additional unidentified proteins^[6]^. Tafazzin can also sense mitochondrial membrane curvature and play a direct role in cristae reorganization and stability^[34,35]^. However, Tafazzin is primarily known for the synthesis of mature cardiolipin via promoting cardiolipin acyl chain remodeling, and is the characteristic lipid found in mitochondrial inner membranes. Cardiolipin is associated with many of the complexes of oxidative phosphorylation (OxPhos) and mitochondrial enzymes involved in ATP production, thereby stabilizing OxPhos supercomplexes. Mitochondrial supercomplexes are assemblies of individual respiratory chain complexes colocalized with cardiolipin found on the inner mitochondrial membrane^[36]^, and increased content of supercomplexes facilitates ATP synthesis^[2,37–40]^. Consistent with this, loss of cardiolipin in patients or in models of BTHS leads to mitochondrial shape irregularities (e.g., swollen, collapsed cristae, honeycomb-like formations, aggregates)^[3,4,41–46]^, decreased mitochondrial maximal oxygen consumption/ATP generating capacity^[8,9,47–57]^, decreased mitochondrial efficiency as defined as phosphate-to-oxygen ratio^[45,56]^, increased apoptosis, and either no change^[48,51,58]^ or increase^[48,52,53,57,59,60]^ in superoxide production. Moreover, in addition to cardiolipin-deficient impairment of OxPhos, several BTHS models also exhibit defects relating to the intermediary metabolism of fatty acids, carbohydrates, ketones, and amino acids^[61]^. Thus, as these alternative fuel substrates are less efficient, ATP production may also be indirectly compromised via upstream metabolic disturbances.

In addition to direct effects on OxPhos enzymes, cardiolipin may also affect the energetic state by its ability to bind with high affinity to the nucleotide metabolism enzymes nucleotide diphosphate kinase (NDPK-D or nme23-H4 or NME4) and creatine kinase (CK). NME4 has a mitochondrial targeting sequence^[62]^ and is found on the inner mitochondrial membrane tightly bound to cardiolipin^[63]^. NME4 catalyzes the phosphotransfer between guanines and adenines (ATP + GDP ↔ ADP + GTP). CK is abundant within mitochondria and the cytosol, and functions as a spatial and temporal buffer of ATP^[64]^. The octameric mitochondrial isoform of CK binds tightly to cardiolipin^[65]^. CK catalyzes the phospho-transfer of ATP to creatine (ATP + Cr ↔ ADP + PCr). Both NME4 and CK may also transfer cardiolipin between the inner and outer mitochondrial membranes, particularly when affecting apoptosis^[66,67]^.

## ENERGETIC STATE OF BARTH SYNDROME

### Note

References chosen for this section were initially identified in or Scopus with a search for Tafazzin and ATP. We selected all those studies that performed measures of ATP in cells/tissues with mutated Tafazzin or decreased Tafazzin protein amounts.

### Cell studies

Numerous groups have measured ATP and/or ADP in cell culture models of cardiolipin insufficiency, mimicking the BTHS condition. Significantly, using immortalized lymphoblasts from BTHS patients, ATP, ADP, and AMP levels were all increased, which is thought to result from a compensatory increase in mitochondrial content^[68]^. Likewise, ATP is increased when wild-type TAFAZZIN is knocked down in cultured cardiomyocytes or knocked out in mouse embryonic fibroblasts^[53]^. In contrast, ATP levels are decreased in fibroblasts^[44]^, lymphocytes^[69]^, and inducible pluripotent stem cell-derived cardiomyocytes^[70]^ from BTHS patients. Further, ATP is also decreased with *shRNA* knock-down of Tafazzin in rat neonatal ventricular myocytes^[60,71,72]^ and in HeLA cells^[50]^, as illustrated in Table 1. Because loss of cardiolipin has clear inhibitory effects on mitochondrial ATP production, it may be surprising that ATP content is not consistently lower across cell types. Potential explanations could be the different metabolic substrates (often very high glucose *in vitro*), very low energy demand, lack of cross-talk between cell types, and the ability of many cells to vary ATP production between glycolysis and OxPhos. Indeed, since ATP is necessary for cell survival, it must be true that the steady-state ATP production rate must match the ATP use rate. Thus, although ATP is often used as a surrogate marker for mitochondrial dysfunction in BTHS, care should be exercised as there may be many ways that energetics can be disassociated.

#### Mouse models:

In mouse models, it remains unclear whether the doxycycline-inducible *Tafazzin* knock-down (*Taz*^*KD*^) or knock-out (*Taz*^*KO*^) mice mutant models exhibit ATP content anomalies, but ATP synthesis (mitochondrial F0F1-ATP synthase) and ADP-ATP carrier abundance are both decreased^[76,77]^. In a patient-tailored point mutant knock-in (*Taz*^*D75H*^) mouse model of BTHS that expresses a stable mutant protein, ATP is lower in the adult heart tissue, with no change in ADP or AMP levels; therefore, the ADP/ATP ratio is higher in adult point-mutant knock-ins. However, no differences were seen in juveniles, demonstrating that the energetic state becomes unstable with disease progression^[42]^. Moreover, despite *Taz*^*D75H*^ mutants exhibiting total infertility, there was no decrease in ATP, ADP or AMP in mutant testis^[78]^, suggesting that not all knock-in mutant organs are equally energetically challenged. In a different model of cardiolipin deficiency, knock-out of *3-hydroxyacyl-coenzyme A dehydratase* (HACD), ATP content was much lower after an exercise bout in the *HACD* knock-outs versus the wild-type controls^[45]^, suggesting that mitochondrial generation was relatively slowed. Similarly, when CL biosynthesis was interrupted via targeted *mitochondrial PTPMT1 phosphatase* deletion in hearts, ATP synthesis was impaired, resulting in abnormal heart development and neonatal lethality^[79]^. However, no direct measures of the non-contracted skeletal muscle energetic state (e.g., ATP and ADP) have been reported in any of the mouse models of BTHS.

### BTHS patients

Given there is only a small number of BTHS patients, the difficulty in obtaining tissue samples from patients with growth insufficiency, and the high incidence of stillbirths and prenatal loss that are associated with BTHS^[80]^, it is not surprising there are no direct measures of the energetic state in heart or skeletal muscle of BTHS patients. However, non-invasive MRS has been used to measure PCr and Pi in heart and skeletal muscle^[8,81]^. This technique does not provide absolute quantification; instead, ADP and Pi are calculated using the CK equilibrium constant^[14]^, assuming that ATP content is normal. In the hearts of both children and adults with BTHS, the [PCr]/[ATP] ratio is lower, suggesting that the ADP/ATP ratio is increased, i.e., a worse energetic state^[8,81]^. In resting skeletal muscle, in children (and a trend in adults), the relative [PCr]/[ATP] was greater, suggesting that the ADP/ATP ratio is decreased, i.e., an improved energetic state^[8]^. The finding of opposite directional changes in the ADP/ATP ratio in heart and skeletal muscle of the same BTHS patients supports the notion that loss of cardiolipin does not result in a predictable change in energetic state. Instead, it depends on other factors influencing cellular energetics, such as substrate supply, tissue type, oxygenation, and energy demand (e.g., muscle contractions).

Clinically, this might be important given the recent targeting of BTHS mitochondria with elamipretide^[82]^. It has been shown that *in vivo* mitochondrial ATP production is improved in older adult skeletal muscle after a single dose of elamipretide in a randomized trial^[83]^. Elamipretide, an aromatic, cationic tetrapeptide, works by localizing to the inner membrane, where it binds to cardiolipin to enhance membrane stability and ATP synthesis in several organs, including the heart^[84]^. Encouraging clinical results observed that elamipretide increases mitochondrial respiration, improves electron transport chain function and ATP production, and reduces pathogenic ROS production. Currently, it is unclear whether functional benefit is achieved through an improvement of ATP or ADP/ATP ratio, an interruption of damaging oxidative stress, or other unidentified factors. Since elamipretide binds to and stabilizes cardiolipin, it would be intriguing to test whether elamipretide may function through other mechanisms by comparing treatment in the patient-tailored point mutant *Taz*^*D75H*^ knock-in^[42]^ versus a *Taz*^*KO*^ knock-out^[77]^ mice. Taken together, further studies are required to determine the effect of cardiolipin loss on the energetic state in the muscles of individuals with BTHS. While ATP levels are decreased in some instances, it does seem clear from cell models that despite a well-described and severe decrement in maximal mitochondrial oxygen consumption/ATP generating capacity when cardiolipin is reduced, ATP depletion or a decrease in the energetic state is not obligatory in BTHS. Therefore, despite ongoing pathology, a near-normal energetic status may be maintained, likely due to cellular compensations such as an increase in mitochondrial number or alternative pathways. Of course, this assumes that some basal rate of ATP production can be maintained, as if ATP production is too low to meet basal ATP needs, then ATP levels will rapidly decrease, and the cell will die.

## ENERGETIC STATE IN OTHER MITOCHONDRIOPATHIES

To determine whether the lack of a consistent link between mitochondrial ATP production capacity and cellular ATP content is unique to loss of cardiolipin, we investigated whether other models of mitochondriopathies showed changes in cellular ATP content. Induced pluripotent stem cell (iPSC)-derived neurons from patients with *DNA polymerase gamma, catalytic subunit* (*POLG*)-related mitochondrial DNA depletion syndrome exhibit decreased mitochondrial content and ATP^[85]^. However, in McArdle’s disease patients with different defects in mitochondrial DNA, ATP levels were not different in skeletal muscle^[86]^. In a mouse model of *Succinate-CoA ligase ADP-forming subunit beta* (an enzyme of the TCA cycle) deficiency, which results in muscle atrophy and muscle weakness in a subset of skeletal muscles, ATP, ADP, and AMP content are normal in those muscles^[87]^. In cell models with pathogenic mitochondrial DNA mutations in *ATP synthase* or mitochondrial-null cells, cytosolic levels of ATP measured by luciferase constructs were no different from wild-type cells^[88]^. Therefore, while impaired OxPhos is an important mediator of ATP production, it is not the sole determinant of steady-state ATP content.

## OUTLOOK

BTHS is a devastating genetic condition caused by mutations in *TAFAZZIN*, which result in limited capacity for mitochondria to produce ATP from ADP. ATP content varies depending on the energetic state of the cell, and the specific cell types and organs being examined, as well as temporal disease progression. Therefore, direct organ-specific measures of ATP are critically important. Many cell models of BTHS and cells from patients with BTHS demonstrate a decrease in relative ATP amount, but others reveal an increase or no change. Unfortunately, most of these studies do not provide ADP measures, which makes it impossible to determine whether the free energy of ATP has indeed been changed or whether the cell/tissue has undergone a phenotypic change that has remodeled the entire adenine nucleotide pool. Therefore, measures of ATP are required, but alone are not sufficient to fully understand the energetic state and thus may limit sweeping interpretations. Further, BTHS and other mitochondrial myopathies are not necessarily characterized by ATP depletion. It is recommended that a more comprehensive approach be used with simultaneous measures of ATP, ADP, and AMP.

## Figures and Tables

**Table 1.** Variance of ATP levels and ADP/ATP ratio measurements in diverse BTHS model systems

Species	ATP level	ADP/ATP	Genetic alteration	Tafazzin protein	Cell type/organ	Reference

*In vitro* cell culture models						
Human	Down	NR	*TAZ^517delG^, TAZ^328T>C^* patients	NR	iPSC-derived CMs	[70]
Human	Up	Unchanged	*TAZ^G197E^, TAZ^I209D^* patients	None	lymphoblasts	[68]
Human	Down	NR	BTHS patients	None	lymphocytes	[69]
Human	Down	NR	*shTAZ* knock-down	Reduced *wt* TAZ	HeLa cervical cancer	[50]
Human	Down	NR	*TAZ^170G>T^, TAZ^140-152del13^*	NR	Fibroblasts	[73]
Mouse	Down	NR	*Taz^KD^* knock-down	Reduced *wt* TAZ	C2C12 skeletal muscle	[60]
Mouse	Up	NR	*Taz^G193V^* knock-in	None	CMs	[53]
Mouse	Up	NR	*Taz^KO^* knock-out	None	MEFs	[53]
Rat	Down	NR	*shTaz* knock-down	Reduced *wt* Taz	Neonatal ventricle CMs	[60,71]
Rat	Down	NR	*shTaz* knock-down	Reduced *wt* Taz	Neonatal ventricle fibroblasts	[72]
Rat	Unchanged	NR	*Taz* knock-out	None	C6 glioma cells	[74]
Yeast	Unchanged	NR	^Δ^*taz1* knock-out	None	*S. cerevisiae*	[75]
*In vivo* organs						
Human	NR	Up*	BTHS (*n* = 6–14) patients	NR	Adult, juvenile hearts	[8]
Human	NR	Down*	BTHS (*n* = 6–14) patients	NR	Adult, juvenile calf muscle	[8]
Mouse	Unchanged	Unchanged	*Taz^D57H^* knock-in	Mutant Taz present	Juvenile ventricles	[42]
Mouse	Down	Up	*Taz^D57H^* knock-in	Mutant Taz present	Adult ventricles	[42]

NR: Not reported; *wt*: wild-type

* Calculated from PCr/ATP ratio *in vivo*.

## Data Availability

Not applicable.
